# Rain splash-mediated dispersal of *Escherichia coli* from fecal deposits to field-grown lettuce in the mid- and south Atlantic U.S. regions is affected by mulch type

**DOI:** 10.3389/fpls.2024.1370495

**Published:** 2024-03-19

**Authors:** Adam L. Hopper, Claire L. Hudson, Diksha Klair, Qiao Ding, Zhujun Gao, Aprajeeta Jha, Austin Bryan, Rohan V. Tikekar, Timothy Coolong, Laurel L. Dunn, Shirley A. Micallef

**Affiliations:** ^1^ Department of Plant Science and Landscape Architecture, University of Maryland, College Park, MD, United States; ^2^ Department of Nutrition and Food Science, University of Maryland, College Park, MD, United States; ^3^ Department of Food Science and Technology, University of Georgia, Athens, GA, United States; ^4^ Department of Horticulture, University of Georgia, Athens, GA, United States; ^5^ Center for Food Safety and Security Systems, University of Maryland, College Park, MD, United States

**Keywords:** preharvest food safety, fecal contamination, lettuce contamination, bacterial dispersal, rain splash, harvest buffer zones, mulches, enteric pathogens on plants

## Abstract

**Introduction:**

Wildlife feces can contaminate vegetables when enteric bacteria are released by rain and splashed onto crops. Regulations require growers to identify and not harvest produce that is likely contaminated, but U.S. federal standards do not define dimensions for no-harvest zones. Moreover, mulching, used to retain soil moisture and maximize crop yield may impact rain-mediated bacterial dispersal from feces.

**Methods:**

To assess *Escherichia coli* dissemination from a fecal point source to lettuce grown on various mulches, lettuce cv. ‘Magenta’ was transplanted into raised beds with plastic, biodegradable plastic, straw, or left uncovered at field sites in Maryland and Georgia. Eleven days post-transplant, 10 g of rabbit manure spiked with ~8 log CFU g^-1^ *E. coli* were deposited in each bed. One day following natural or simulated rain events, lettuce was sampled along 1.5 m transects on either side of fecal deposits. Lettuce-associated *E. coli* was semi-quantified with an MPN assay and dependence on fecal age (stale or fresh), lettuce age (baby leaf or mature head), distance from point source, mulch and post-rain days were statistically evaluated.

**Results:**

Distance (*p*<0.001), fecal age (*p*<0.001) and mulch (*p*<0.01) were factors for *E. coli* transfer from point source to lettuce. The highest and lowest *E. coli* estimates were measured from lettuce grown on biodegradable plastic and straw, respectively, with a 2-log MPN difference (*p*<0.001). Mulch and distance were also significant factors in *E. coli* recovery 3 days post-rain (both *p*<0.001), where plastic mulches differed from bare ground and straw (*p*<0.01). For all treatments, fewer *E. coli* were retrieved from lettuce at 0.3 m, 3 days post-rain compared to 1 day (*p<*0.001). Fitting the data to a Weibull Model predicated that a 7-log reduction in *E. coli* from fecal levels would be achieved at 1.2-1.4 m from the point source on plastic mulches, 0.75 m on bare soil (*p*<0.05) and 0.43 m on straw (*p<*0.01).

**Discussion:**

Straw and bare ground limited rain-mediated *E. coli* dispersal from feces to lettuce compared to plastic mulches. Fecal age was negatively associated with *E. coli* dispersal. These findings can inform harvesting recommendations for measures related to animal intrusion in vegetable production areas.

## Introduction

Contamination of leafy vegetables with enteric pathogens during the pre-harvest phase can result in major foodborne illness outbreaks leading to human infection, death and substantial economic losses (Berger et al., 2010; Coulombe et al., 2020; IFSAC, 2022). Hazards emanating from the field pose a major challenge due to the sporadic nature of enteropathogen occurrence and transmission via irrigation water, biological soil amendments and wildlife feces. Wildlife can serve as reservoirs or transitory hosts (Renter et al., 2001; Martínez et al., 2011; Siembieda et al., 2011), and consequently pathogens can be disseminated to crop surfaces by direct contact with scat or through bacteria leached from droppings via water splash from over-head irrigation or rainfall (Franz and van Bruggen, 2008; Lee et al., 2019). Foodborne illness outbreak tracebacks to pathogens from wildlife feces include *Escherichia coli* O157:H7 from wild pigs linked to spinach (Jay et al., 2007) and *Campylobacter jejuni* from cranes linked to snow peas (Kwan et al., 2014).

Knowledge of the mechanisms of pathogen dissemination at the field level is crucial for development of best practices for growers to implement during harvest of leafy vegetables to minimize crop contamination from animal feces. A few studies have investigated the transmission of *E. coli* from animal feces to crops by irrigation water splash in the field. These studies quantified the bacterial transmission from fecal deposits onto romaine lettuce under sprinkler irrigation on the west coast and northeastern U.S., finding that *E. coli* transfer was dependent on the age of the fecal deposit before irrigation and the distances between lettuce and feces or the sprinkler head (Atwill et al., 2015; Weller et al., 2017; Jeamsripong et al., 2019). No data exists for the mid- and south Atlantic states of the U.S., where farming scale, crop management practices and climate differ.

The Food Safety Modernization Act (FSMA), Produce Safety Rule Title 21 CFR Part 112 (Food and Drug Administration, 2015) does not provide specific standards on how to handle the discovery of animal excreta in the field at the time of harvest but states that growers must identify and abandon produce that is likely to be contaminated with animal feces (Subparts I § 112.83 and K § 112.112). More specific guidance for leafy vegetable growers regarding detection of animal feces in crop production areas at the time of harvest comes from the California Leafy Greens Marketing Agreement (LGMA) (California LGMA, 2023), which establishes a no-harvest buffer zone, a minimum 1.52 m (5 feet) in radius, around the fecal deposit.

Since bacterial transmission can be affected by field management practices and weather patterns, there is a need for region specific data to determine the effectiveness and adequacy of the LGMA guideline, when adopted outside of California. The Atlantic states typically receive well over 1,000 mm of rain annually while the west coast experiences frequent periods of drought. The likelihood of rain splash mediated transmission of enteric pathogens from animal excreta is therefore higher in the mid- and south Atlantic states. Many growers in these regions also incorporate row covers or mulching into farming practices to suppress weeds, preserve soil moisture and regulate soil temperatures (Kelly et al., 1995). Various forms of mulches are used, including black or white plastic materials, biodegradable plastic polymers, paper, straw, grass clippings and wood chips (El-Beltagi et al., 2022). Mulching has been investigated for effects on soil microclimate and soil and phyllosphere microbial dynamics (Xu et al., 2016; Micallef et al., 2023), but mulch effects on bacterial dispersal patterns are not well characterized.

The need for region-specific research on dispersal of bacteria from animal fecal deposits in crop production areas onto leafy green crops is important to fully understand the food safety risks that surround wildlife intrusion and how these risks can be managed. Therefore, the goal of the present study was to investigate the dissemination of generic *E. coli* from a fecal point-source onto lettuce during field production, as mediated by rain-splash or simulated rain-splash. We aimed to assess the effectiveness of a 1.52 m no-harvest buffer zone in the mid-Atlantic and southeastern U.S. and address the dearth of data for this region. The study was conducted over two years in open-field environments on loose-leaf lettuce at two harvestable stages, baby leaf and mature head lettuce. Trials were conducted to evaluate how distance from fecal point-source, plant age, feces age and mulch type would impact rain-mediated bacterial dissemination from feces. Field residence time of fecal deposit and sampling time post-rain were evaluated to better understand temporal scales for bacterial persistence in fecal deposits and following transfer to the crop. The findings from this study contribute new insights on rain-splash mediated dispersal of *E. coli* onto edible greens and can inform metrics related to harvesting of leafy greens when fecal contamination is detected in the field at the time of harvest.

## Methods

### Plant material and field design

The field trials were conducted in the states of Maryland and Georgia, U.S. The Maryland field plot was located at the University of Maryland Wye Research and Educational Center, Queenstown, MD and the Georgia field trial was conducted at the University of Georgia Horticultural Research Farm, Watkinsville, GA. Loose-leaf lettuce cv. ‘Magenta’ untreated seeds (Johnny’s Selected Seeds, Winslow, ME) was sown in flats under greenhouse conditions and transplanted into the field 3 weeks post-germination. In the field, plants were maintained using conventional farming practices and irrigated with drip lines below the mulch and soil surface. Plants were irrigated for approximately 1 h daily after establishment. No herbicides were applied prior to planting and no pesticides were applied during growth. Weeds were removed by hand.

**Figure 1:**
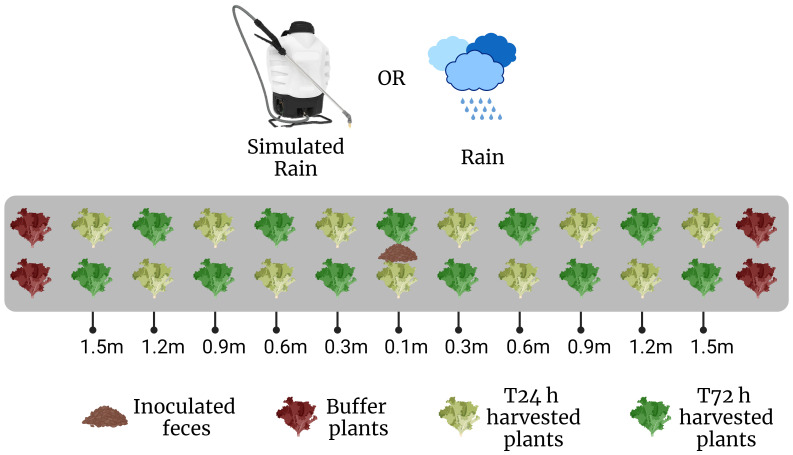
Field layout for one bed, planted with ‘Magenta’ lettuce (n=22 per bed). Lettuce seedlings were transplanted 0.3 m apart in double rows as depicted. Inoculated rabbit scat was placed in the middle of each bed 0.1 m away from two rows of lettuces. Sampling was conducted after a rain event at 0.3 m intervals along the lettuce transect in a staggered, diagonal fashion on either side of the scat. (Created with BioRender.com).

The general scheme for each trial was to place fecal deposits in the middle of 3 m long lettuce beds before rain, followed by lettuce sampling 1 or 3 days after rain, to evaluate the amount and distance of *E. coli* dispersal from the fecal deposits to the lettuce plants (**Figure 1**). Details of each trial are given in **Table 1**. The field plot consisted of drip-irrigated raised beds laid with plastic mulch. Lettuce was planted 0.3 m apart in double rows with individual treatment beds consisting of 11 pairs of lettuce plants, with 3 additional pairs as a buffer zone on either end of each treatment bed (**Figure 1**). Twenty-eight randomized beds were dedicated to 4 different treatments (n=7/treatment). These were stale feces (field placement 3 days before rain event) and baby leaf lettuce (10 days after transplanting), and fresh feces (field placement 1 day before rain event) and baby leaf lettuce (Trial 1A); stale feces and mature lettuce (36 days after transplanting), and fresh feces and mature lettuce (Trial 1B). Sampling of lettuce occurred 1-day post-rain event in Trial 1A and Trial 1B plots. A similar trial with fresh feces using a baby leaf lettuce crop was conducted in Georgia (Trial 2), with the difference that lettuce was planted on bare soil with no ground cover.

**Table 1:** Specifications for each field trial conducted in Maryland and Georgia.

Specifications	Trial 1A	Trial 1B	Trial 2	Trial 3	Trial 4
Location	Maryland	Maryland	Georgia	Maryland	Georgia
Feces age/ Interval between placement and rain event	Stale/72 h Fresh/1 h	Stale/72 h Fresh/1 h	Stale/72 h Fresh/1 h	Fresh/1 h	Fresh/1 h
Log CFU/10g feces (stale; fresh)	7.6; 8.3	8.1; 8.3	8.4; 8.6	8.8	8.8
Lettuce stage	Baby leaf	Mature head	Baby leaf	Baby leaf	Baby leaf
Rain event	Simulated	Natural (6 mm)	Simulated	Natural (11 mm)	Simulated
Mulch Types	Black plastic	Black plastic	Bare Ground	Black plastic; biodegradable plastic; straw; bare ground	White plastic; straw; bare ground
Sampling time (h post-rain event)	24	24	24	24; 72	24

Two additional trials were conducted in Maryland and Georgia using fresh feces in baby leaf lettuce plots (Trials 3 and 4). A randomized plot with different mulch treatments was set up, including biodegradable black plastic, black plastic, straw and bare soil (no mulch control) in the Maryland trial, and white plastic, straw and bare soil in the Georgia trial (**Table 1**).

### Manure preparation

A frozen stock of *E. coli* TVS353 isolated from irrigation water (Salinas Region, CA, USA) (Tomás-Callejas et al., 2011) was streaked on tryptic soy agar (TSA) (Difco, Sparks, MD) with 80 µg·µL^-1^ rifampicin (TSAR; Difco, Sparks, MD) and incubated at 37°C for 24 h. A single colony was transferred into tryptic soy broth with rifampicin (TSBR) and incubated at 37°C with shaking for 24 h. On the morning of the trial, the culture was spun down at 4000 g for 10 min at room temperature and supernatant discarded. The cell pellets were resuspended in 10 mL of 0.1% peptone water (PW) (Difco, Sparks, MD) and 1 mL of the resuspended culture fully incorporated into 10 g of rabbit feces (The Classic City Rabbitry, Hull, GA) in a Whirl-Pak bag (Nasco Whirl-Pak™, Madison, WI) by hand massaging the bag for 90 s, for a final concentration of ~8 log CFU·g^-1^. Manure bags were placed on ice to be transported to the field for placement within 2 h. Each inoculum and a sample from a representative bag of spiked rabbit feces were plated for accurate quantification of *E. coli* used in each trial. Additionally, an uninoculated bag of rabbit manure was plated onto TSAR to confirm the absence of rifampicin resistant microflora in the feces.

### Field application of fecal deposits and rain/simulated rain events

Ten days (baby leaf lettuce) and 36 days (mature lettuce) after transplanting, manure bags were emptied in the middle of each raised bed between the two rows of lettuce at 8 m intervals, 3 days (stale) and 1 hour (fresh) before natural/simulated rain. While natural rain events were preferable, rain was simulated on dry days, to keep the crop and study schedule the same across regions over the two years. To simulate a moderate rainfall (based on the American Meteorological Society observational rain intensity classification (https://glossary.ametsoc.org/wiki/Rain), a rate of 4 mm·m^2^·h^-1^ in a 0.1×0.1 m^2^ area was employed by spraying 40 mL of sterile distilled water using a low volume fan nozzle at a rate of 800 mL·min^-1^ (40 mL·3 s^-1^) from a backpack sprayer (Field King Max 190348 Back Pack Sprayer, New York Mills, NY). To ensure uniform pressure during each application, the piston was pumped until it was completely tightened before each spray. The nozzle tip was held at approximately 0.5 m above the manure with the water flow perpendicular to the ground. When naturally occurring rainfall was forecast, rain simulation was not conducted (**Table 1**). Relevant weather data were collected from weather stations located at each experiment station (**Table 2**).

**Table 2:** Temperature in °C and precipitation in mm recorded during field trials.

	Trial 1A	Trial 1B	Trial 2	Trial 3	Trial 4
Feces condition	Stale/Fresh	Stale/Fresh	Stale/Fresh	Fresh	Fresh
Rain event (mm)^a^	0.25/0	0/6	3	11	0
Cumulative rainfall (mm) between feces placement and rain event (not including rain event)^b^	0.25/0	0/0	12/0	0	0
Mean max temp (°C) post-feces placement^c^	26/26	28/27	24/21	25	21
Mean min temp (°C) post-feces placement^c^	13/11	18/19	17/17	18	7
Mean max/min temp (°C) between 24 h and 72 h sampling	–	–	–	20/14	–

a Rain event refers to natural precipitation or simulated rain directly after placement of feces and used to assess E. coli dispersal. Rain was simulated when natural precipitation was not forecast.

b Time between feces placement and rain event was 24 h for trials using fresh feces and 72 hours for trials using stale feces.

c For stale feces trials, temperature is the 72-h mean after feces were placed in the field. For fresh feces trials, temperatures on the day of feces placement are given.

### Lettuce sampling

Plants were sampled 24 hours after each rain event. This represented a 24-hour harvest wait period following rain. Starting from the outermost distance from the contamination point source, 2 plants from opposite sides of the fecal deposit were sampled at 0.1, 0.3, 0.6, 0.9, 1.2 and 1.5 m along two transects from the feces. In Trials 1A, 1B and 2, there were 14 total beds per plant age: 7 stale and 7 fresh feces (**Figure 1**). Except at the 0.1 m distance where both plants next to fecal deposit were collected, plants were sampled in a diagonal pattern on either side of the point source, to account for variability in wind direction. Whole plants were gripped with forceps and cut with scissors, then placed in sterile Whirl-Pak bags. Gloved hands, forceps and scissors were sprayed with 70% ethanol between each sampling. Bags were placed on ice for transport to the lab and processed within 4 h. In Trials 3 and 4, there were 7 beds per treatment, sampled 24 h after the rain event. In Trial 3, lettuce was additionally sampled 72 h after the rain event at all distances except 0.1 m. Negative controls were sampled prior to fecal application and conducted in triplicate.

### Lettuce processing

For baby leaf lettuce, two whole plants were combined in one Whirl-Pak bag and diluted 1:5 (w/v) with phosphate buffer solution (PBS) for enumeration following a 4- tube modified Most Probable Number (MPN) method. For mature lettuce, two heads of lettuce from each distance were aseptically chopped and mixed thoroughly and a 30 g subsample processed in 120 mL PBS in a fresh Whirl-Pak bag. Bags were stomached in a stomacher (Seward Stomacher 400 Circulator Lab Blender, Vernon Hills, IL) at 230 RPM for 45 s and 1 mL of homogenate added to all 4 rows of the first column of a 96-well plate containing 1 mL of 2x TSBR. The well contents were mixed via pipette aspiration and serial dilutions were made by transferring 200 μl from each well into 1.8 mL of 1x TSBR in each subsequent well for 6 total dilutions. Plates were incubated at 37°C for 24 h, then suspensions streaked onto TSAR and incubated at 37°C for 24 h. Plates were recorded for presence/absence of *E. coli* growth. *E. coli* confirmation for positive growth was conducted by streaking the TSAR suspension onto MacConkey agar plates with rifampicin (MACR) (Difco, Sparks, MD).

### Statistical analysis and modelling

The EPA most probable number (MPN) calculator (US, EPA, 2013) was used to estimate *E. coli* levels in each sample. MPN value estimates were computed using probability formulas and log transformed to report log MPN/sample, with sample representing 2 whole plants for baby leaf lettuce and 30 g of chopped leaves for mature lettuce heads. Log MPN/sample data were fitted to a standard least squares model with Tukey’s Honestly Significant Difference (HSD) test in JMP Pro ver. 15.2 (SAS Institute Inc., Cary, NC) to assess significant differences due to feces age, plant age, distance from fecal point source, mulch type and sampling time post rain event. *E. coli* log MPN reduction from level in inoculated feces was calculated and the Weibull Model was fitted to Trial 3 data, generating model parameters that were used in a nonlinear regression function in JMP using the following equation (Ding et al., 2023):


$$log\left(\frac{N_{d}}{N_{0}}\right)=-\frac{1}{2.303}{\left(\frac{d}{\alpha }\right)}^{\beta }$$

where α is a scale parameter describing the characteristic distance (m), where log (N_d_/N_0_)=0.434 (or N_d_/N_0_=e^-1^), and β is a shape parameter of the decay curve. Data from the following experimental conditions were used: bacterial levels recovered from lettuce 24 h post rain event, from lettuce grown under different mulch conditions, and when fresh scat was employed to simulate a rain-mediated contamination event. The root mean squared error (RMSE) and the RMSE-observations standard deviation ratio (RSR) were calculated to determine model goodness of fit (Moriasi et al., 2007). RSR was calculated for each model as the ratio of the RMSE and standard deviation (SD) of observations using the following equation:


$$\;RSR=\frac{RMSE}{SD}$$

Using parameters generated from the Weibull Model, the distance (d, m) needed to reach x-log reduction (x= 3, 4, 5, 6, 7) was computed for each treatment using the following equation (Ding et al., 2023):


$$d_{x}=\alpha {\left(2.303x\right)}^{\frac{1}{\beta }}$$

An analysis of variance (ANOVA) with HSD was employed to determine differences between the Weibull estimated distance required to reach each log reduction.

## Results

### Splash-mediated *E. coli* transfer from fresh and stale feces to baby leaf and mature lettuce crops

**Figure 2:**
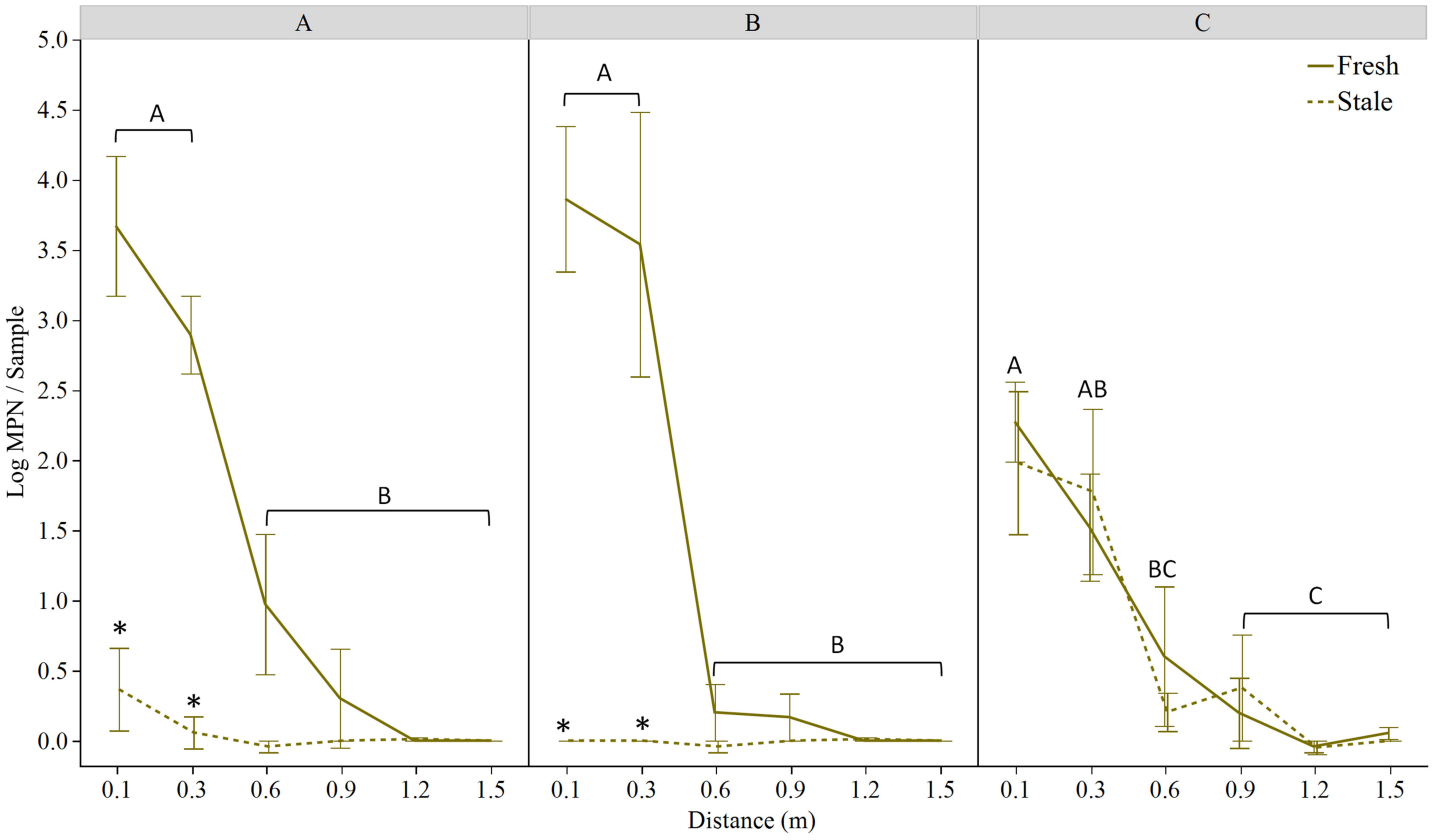
Log MPN *E. coli* TVS353 retrieved from ‘Magenta’ lettuce at the stage of **(A)** baby leaf and **(B)** mature head grown on black plastic mulch in MD, and **(C)** baby leaf ‘Magenta’ lettuce grown on bare ground in GA, 24 h after feces placement in the field and a simulated **(A, C)** or natural **(B)** rain event. Solid and dashed lines represent data obtained from lettuce beds receiving fresh and stale inoculated rabbit scat, respectively. Asterisks indicate significant differences between fresh and stale treatments at each distance within each panel. In **(A, B)**, capital letters indicate significant differences among distances for the fresh feces trial using LSmeans Tukey HSD (*p*<0.05). Capital letters shown in **(C)** indicate differences for both the fresh and stale trials, which exhibited a similar trend.

No rifampicin resistant *E. coli* was detected in either the rabbit scat prior to inoculation with TVS353 or on the lettuce plants prior to the experiments. The actual *E. coli* concentrations in field-placed feces (**Table 1**) were determined 2 hours after spiking with *E. coli* and storing the feces on ice, representing travel time to the field. After the rain/simulated rain events, *E. coli* TVS353 was detected in all lettuce trials. As a whole, 27%, 17% and 38% of samples were positive for *E. coli* in Trials 1A, 1B and 2, respectively. Both *E. coli* presence and concentration were inversely associated with distance from the point source. While 64%, 50% and 92% of samples were positive for *E. coli* at the 0.1 m distance, respectively, 0% in Trials 1A and 1B and 15% in Trial 2 were positive at 1.5 m. Distance from the point source was a significant factor for *E. coli* levels at both the Maryland and Georgia (*p*<0.001 for both) field sites, whereby *E. coli* MPN estimates decreased as distance from point source increased. Feces age was a factor at the Maryland site only (*p*<0.001), where more *E. coli* dispersal was observed from fresh feces (**Figure 2**). The starting *E. coli* concentration was within 0.7 log CFU·g^-1^ in Trial 1A, and within 0.2 log CFU·g^-1^ in Trials 1B and 2, for stale and fresh feces. We found that starting at the 0.1 m distance, dispersed *E. coli* varied by feces age in Trials 1A and 1B (Maryland), but not Trial 2 (Georgia), and by geography/cultivation practice (Maryland using plasticulture versus Georgia planting on bare ground) (**Figure 2**).

No overall effect of lettuce age was detected on *E. coli* levels recovered at various distances from the fecal point source (**Figures 2A, B**); *E. coli* levels decreased along the 1.5 m transect in both lettuce crops in a similar way. In the Maryland trial, *E. coli* MPNs retrieved from both baby leaf and mature lettuce at 0.1 (*p*<0.001 for both ages) and 0.3 m (*p*<0.05 and *p*<0.001, respectively) away from the fresh fecal point source were higher than levels measured at farther distances. The largest reduction was measured between the 0.3 and 0.6 m distances, with reductions of 1.9 log MPN/sample for baby leaf lettuce and 3.3 log MPN/sample for mature lettuce (**Figures 2A, B**). At the Georgia field site, bacterial estimates declined at a more uniform rate along the transect (**Figure 2C**).

When comparing fresh and stale feces, in the Maryland trial, significantly lower levels of *E. coli* contamination were detected in lettuce beds with stale droppings compared to fresh at sampling distances 0.1 m and 0.3 m (*p*<0.001 for baby leaf and mature plants) only (**Figures 2A, B**). No other differences were detected at 0.6 m and beyond. At the Georgia field site (Trial 2), no differences in the level of contamination from fresh or stale droppings were detected between any of the distances sampled (**Figure 2C**).

### Splash-mediated *E. coli* transfer from feces to lettuce grown under different mulches

**Figure 3:**
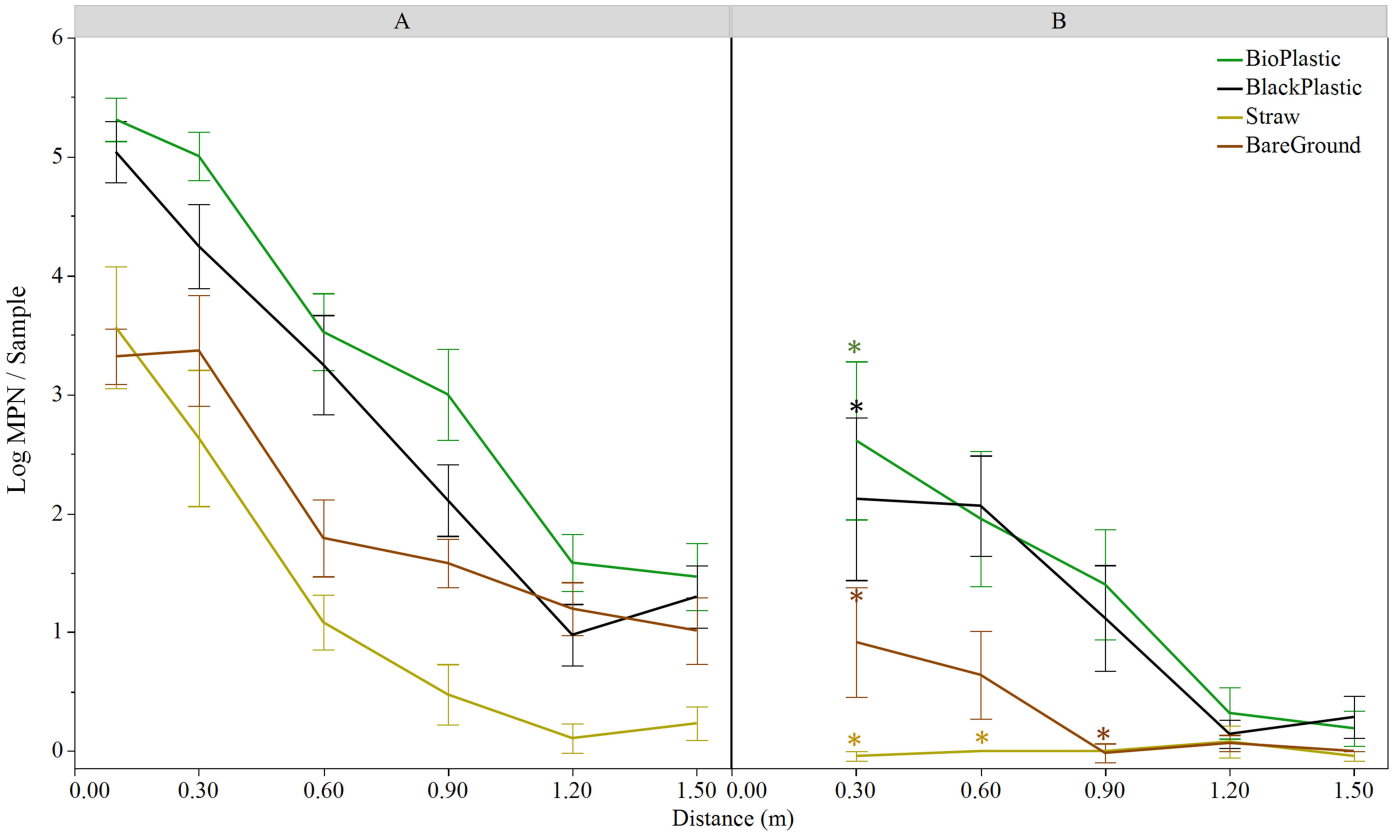
Log MPN/sample of *E. coli* TVS353 retrieved from baby leaf ‘Magenta’ lettuce grown over different mulch covers in Maryland **(A)** 24 h and **(B)** 72 h post feces placement in the field and a natural rain event. Asterisks represent significant differences between 24 h and 72 h sampling time points at each distance using LSMeans and Tukey HSD test (p<0.05). Significant differences (*p*<0.05) among mulches were detected at 0.1, 0.3 and 0.6 m **(A, B)** and 0.9 m **(A)** distances and are reported in the text.

Distinct *E. coli* dispersal patterns were observed between the two field sites in Maryland and Georgia where different cultivation practices were adopted. Hence, to determine whether mulches influenced the degree of bacterial dissemination from a fecal point source to lettuce following a rain event, a second set of trials were conducted to compare a series of ground covers commonly used in each region (**Figure 3**; **Table 3**). As previously recorded, distance was a significant factor for *E. coli* transfer from point source to lettuce in both Maryland (*p*<0.001) and Georgia (*p*<0.001) on all mulches. Moreover, level of dispersal was also affected by mulch type (*p*<0.001 in Maryland and *p*<0.01 in Georgia). In Maryland, *E. coli* MPNs differed significantly among all mulches, with biodegradable black plastic and straw displaying the largest overall difference (2 log, *p*<0.001) (**Figure 3A**). In Georgia, *E. coli* MPNs from white plastic covered beds were significantly higher than both straw and bare ground beds, but the difference was small overall (0.2 log, both *p*<0.05) (**Table 3**). There was also a difference in % positive samples among mulch types and between sites. In Trial 3, straw generated the lowest number of *E. coli* positive samples at 50.6%, while plastic had the highest at 85.7%. In Trial 4, the white plastic mulch treatment generated 26.2% positive samples while straw and bare ground were very low at 2.4%.

**Table 3:** Mean log MPN/sample and standard error of *E. coli* TVS353 retrieved from baby leaf ‘Magenta’ lettuce grown along a 1.5 m transect on different mulches at the Georgia field site.

Mulch Type	Distance (m)					
	0.1	0.3	0.6	0.9	1.2	1.5
White Plastic	1.37 ± 0.57	0.03 ± 0.12	0	0	-0.04 ± 0.04	0
Straw	0	0	0	0	-0.04 ± 0.04	0
Bare Ground	0.01 ± 0.01	0	0	0	0	0

Comparable patterns of diminishing *E. coli* recovery with distance from the point source were measured for every mulch type 24 h after a rain event, as had been observed in the previous trial on black plastic and bare ground. However, this varied by mulch type (**Figure 3A**). All along the 1.5 m transects, straw and bare ground restricted dispersal of *E. coli* from the fecal point source, when compared to the black plastic mulches (**Figure 3A**; **Table 3**). Straw and bare ground differed from biodegradable plastic at 0.1, 0.3 and 0.6 m (*p ≤* 0.05), and straw differed to both plastics also at 0.9 m (*p ≤* 0.05). Differences with black plastic were also detected with bare ground at 0.1 m (*p*<0.05) and straw at 0.6 m (*p*<0.001) (**Figure 3A**).

To determine the behavior of *E. coli* populations over time once transferred to the lettuce crop, an additional sampling was conducted 72 h post feces placement in the field under each mulch treatment at the Maryland site (**Figure 3B**). Similarly, mulch and distance from point source were both factors in *E. coli* MPNs at 72 h (both *p*<0.001). In the 72-h trial, both black plastic mulch types differed from bare ground and straw (*p*<0.01), but the latter two were not different from each other. Different *E. coli* levels were measured between straw and both plastics at the 0.3 m and 0.6 m distances (*p ≤* 0.01). No differences by mulch type were detected at 72 h at 1.2 m and 1.5 m distances, where *E. coli* levels had dropped to or below 0.15 log MPN/sample.

Time post feces placement was also a significant factor for *E. coli* levels (*p*<0.001). Less *E. coli* was retrieved from lettuce 72 h post rain event at 0.3 m compared to the 24 h sampling time for each mulch type (*p ≤* 0.05 for all four mulches) (**Figure 3B**). No significant differences between sampling times were detected for *E. coli* retrieved at distances >0.3 m for any mulch, except for straw and bare ground (*p*<0.05) when mulches were analyzed separately (**Figure 3B**).

*E. coli* retrieved from lettuce grown under different mulch conditions at the Georgia field location (Trial 4) were below the limit of detection for straw and bare ground. However, lettuce grown on plastic mulch showed steep declines of *E. coli* contamination at 0.1 m and 0.3 m compared to levels at the point source (**Table 3**).

### Modelling *E. coli* decline along a lettuce transect as a factor of distance from a fresh fecal point source

**Figure 4:**
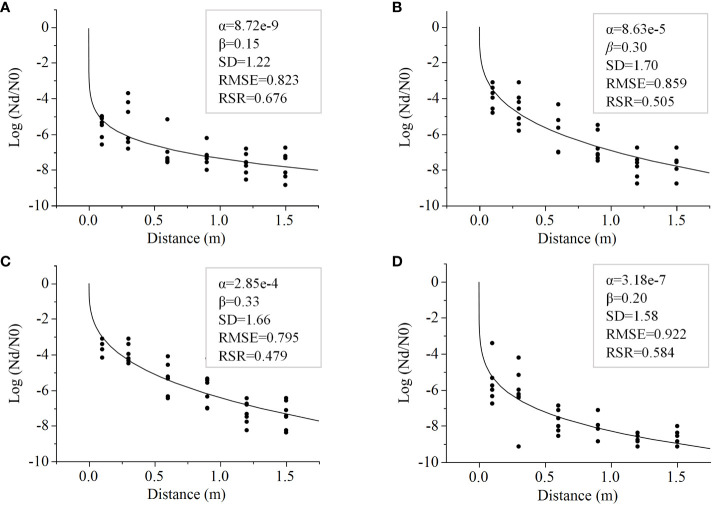
Data from *E. coli* TVS353 reduction from the inoculum in feces (log N_d_/N_0_) with increasing distance from the fecal point source, retrieved from lettuce grown with different mulch types, **(A)** bare ground, **(B)** black plastic, **(C)** biodegradable plastic and **(D)** straw, were fitted to the Weibull Model. Model parameters (α and β) are provided in the boxes along with standard deviations (SD) and two measures of goodness of fit; Root mean square error (RMSE) and the RMSE-observations standard deviation ratio (RSR).

To predict at what distance along a transect *E. coli* levels would dissipate by 3-7 log reductions from the levels in feces, the Weibull Model was fitted to data from Trial 3 obtained 24 hours post-rain event from lettuce grown on various mulches. The fitted Weibull Models (RSR ≤0.68) facilitated the evaluation of *E. coli* dispersal through the fitted α and β parameters (**Figure 4**). The Weibull Model goodness of fit was evaluated as described by Moriasi et al. (2007), RSR values are considered “satisfactory” when the computed RSR is< 0.70. Each model generated by mulch has an RSR< 0.68 and an RMSE of ≤0.9 (**Figure 4**).

**Figure 5:**
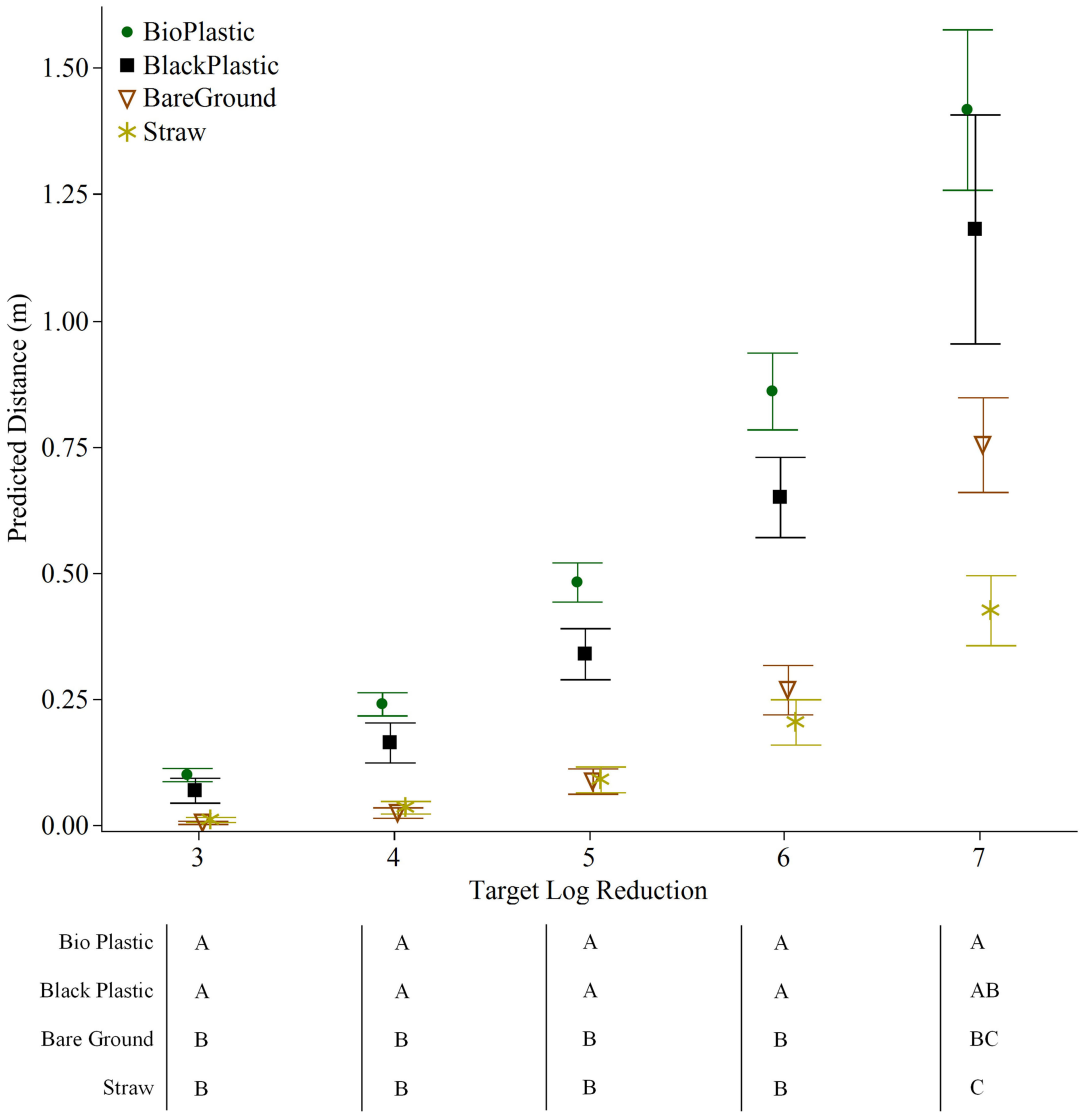
Mean distance and standard error predicted using the Weibull Model to estimate 3-7 log reductions of *E. coli* TVS353 retrieved from baby leaf lettuce grown on different mulch conditions in the field 24 h post placement of fresh feces and following a rain event. Table represents one way analysis of variance (ANOVA) with Tukey HSD test (*p*<0.05) using predicted distances. Mulch treatments not connected by the same letter indicate significant differences for Weibull Model predicted distances for each log reduction.

The Weibull Model clearly indicated similarities in the pattern of *E. coli* spread over a 1.5 m distance between bare ground and straw, and between plastic mulches (biodegradable plastic and black plastic). The predicted distance required to reduce *E. coli* by 5 log was significantly farther for both plastic mulches (0.5 m for biodegradable plastic and 0.3 m for black plastic) compared to bare ground or straw mulch (both 0.1 m) (*p*<0.001 for both) (**Figure 5**). The model for a 7-log reduction, on the other hand, favored straw as the mulch that best attenuated *E. coli* dispersal, followed by bare ground. The predicted distances were 0.4 m and 0.7 m, respectively, compared to plastic mulches that generated a predicted distance of >1 m (1.4 m and 1.2 m for biodegradable plastic and plastic, respectively; straw versus both plastics, *p*<0.01; biodegradable plastic versus bare ground, *p*<0.05) (**Figure 5**). Significant differences were never detected between bare ground and straw, or between biodegradable plastic and black plastic in any of the modelled log reductions of 3-7 log (**Figure 5**).

## Discussion

Feces excreted by animals intruding in vegetable cultivation areas close to the time of harvest may raise the risk of crop contamination with foodborne pathogens, especially in the event of rain that can facilitate bacterial release from fecal matter (Moriarty and Gilpin, 2014). In the present study, dispersal of generic *E. coli* from a fecal point source following natural and simulated rainfall was successfully demonstrated at two sites in the Atlantic US region, in Maryland and Georgia. The level of generic *E. coli* TVS353 transferred to lettuce was highest within 0.3 m of the fecal deposits and tapered off with distance from the fecal point sources of fresh and stale feces.

Trials employing natural and simulated rain events generated comparable data trends, such that we could attribute differences we measured to the tested variables. Recovery of *E. coli* from lettuce in close proximity to fresh feces following a rain event was significantly higher than stale feces in the Maryland trial. While a difference of 0.7 log CFU·10 g^-1^ of feces in the starting inoculum was determined for stale and fresh feces for baby lettuce in Trial 1A, the difference for mature lettuce in Trial 1B was only 0.2 log CFU·10 g^-1^. Therefore, starting inoculum did not appear to be the major driver for this discrepancy in *E. coli* MPN estimates between stale and fresh plots. In agreement with our trial, previous field studies found that time of rabbit feces deposition in the field was negatively associated with *E. coli* transfer to a crop following irrigation (Atwill et al., 2015; Weller et al., 2017). Dry feces that appear to have formed a crust and lack moisture may require more force from rain splash to detach the bacteria from fecal matter, as was hypothesized by Atwill et al. (2015), and studied with nematode larvae (Grønvold, 1984) and fecal coliforms in stale cow manure (Kress and Gifford, 1984). In the Georgia trial (Trial 2), however, stale feces generated a higher level of dispersal than measured in Maryland, where bacterial transfer was minimal. Differential *E. coli* dispersal from stale feces could be dependent on processes such as bacterial survival in the rabbit feces paired with exposure to environmental conditions. Stale feces were present in the field for 72 h prior to the experimental rain event. The Georgia plot (Trial 2) received 12 mm of natural rain between the time of stale feces placement and the time of fresh feces placement (24 h prior to the experimental rain event), while Maryland Trial 1A received 0.25 mm and trial 1B received none. Feces wetting would favor bacterial survival, growth and dispersal, while solar inactivation and desiccation could account for bacterial die-off or transition to non-culturable states. Hence, we hypothesize that the differences detected among Trials 1A and 2 in the baby leaf stale feces plots can be explained by the variability in the amount of rain received between time of stale feces placement and time of fresh feces placement. Moreover, the black plastic used in the Maryland plots retains more heat than bare soil (Amare and Desta, 2021), on which lettuce was grown in Georgia. In addition, we note that rain-activated release of *E. coli* from aged sheep fecal matter has been reported (Moriarty and Gilpin, 2014). The metabolic activity of *E. coli* O157:H7 was higher in sheep feces than cow feces and bacterial leaching from sheep feces following a simulated rainfall was greater than from cattle feces (Williams et al., 2008), suggesting that the composition of feces from different animal species could also be a factor in *E. coli* survival and release. Based on our findings, we conclude that in the absence of wetting, the likelihood of bacterial transmission from feces to lettuce is reduced the longer feces lie in the field, but future studies should investigate rain-mediated bacterial dispersal from a variety of local wildlife to better understand food safety risks. Finally, although we prepared the starting inoculum used to spike the feces to the same concentration, some discrepancy was detected when *E. coli* was enumerated in feces after fecal deposit preparation. As such, some of the variability between stale and fresh data could be attributed to differences in starting inoculum.

A more in-depth look at the data obtained from the fresh feces plots in Maryland and Georgia revealed that *E. coli* counts at the 0.1 m distance were lower in Trial 2 (Georgia) than those obtained in Trial 1A (Maryland). Indeed, the resulting *E. coli* counts were equivalent to those obtained in the stale feces plot for this site. This further suggests that the stale feces in Georgia were affected by rain, allowing *E. coli* to survive for longer in stale feces. However, we do not have a conclusive explanation for why *E. coli* levels were lower in the Georgia fresh feces plot at 0.1 m compared to Maryland. This may have been a factor of the simulated rain. Although we endeavored to implement the same protocol when applying simulated rain, differences in user application, wind direction and speed, relative humidity and insolation could all affect bacterial counts. Nevertheless, we could not discount the possibility that soil type and ground cover played a role in *E. coli* dissemination to lettuce, since lettuce grown in Georgia was transplanted to bare soil and that in Maryland to plastic-covered beds. We surmised that bare soil would absorb rain droplets laden with leached *E. coli*, effectively attenuating the distance droplets would travel, while water impervious plastic mulches would favor repeated ground contact and rebounding of droplets generated by rain, augmenting distance travelled. A relationship between the physical properties of mulches that affect permeability and splash dynamics has recently been demonstrated (Wang et al., 2023). At each site, commonly used row covers were assessed, with bare ground serving as a no mulch control. While all mulches exhibited a distance gradient in the amount of *E. coli* recovered from lettuce after the rain event in a similar trend to the previous trials, differences by mulches were indeed easily measurable. The various plastic row covers favored *E. coli* dissemination as compared to straw and bare soil, providing evidence that soil and straw might be effective in absorbing splash and mitigating bacterial transfer from a fecal point source. Additionally, the Weibull Model predicted a clear cut-off in estimated distance needed to achieve a 7-log reduction in *E. coli* between plastic and straw or no mulching. In Georgia, where minimal bacterial recovery was obtained in the mulch comparison trial, white plastic mulch still yielded slightly higher bacterial levels than straw and bare ground. We attribute the higher % positive rate obtained in Trial 3, compared to Trial 4, to the substantial experimental rain event (11 mm) that Trial 3 was based on. The data suggest that rain intensity is a determinant in need of further investigation. Rain intensity is positively related to bacterial release from manure (Blaustein et al., 2016). Therefore, it is possible that moderate rain intensities would affect bacterial dispersal, as may have occurred in this study, while heavier rainfall may have the effect of washing away microbiota from the plant phyllosphere, as has been previously suggested (Cevallos-Cevallos et al., 2012; Xu et al., 2016). The effect of rain rate on the complex dynamics of bacterial release from feces, transmission to crops as influenced by ground cover, and bacterial persistence in the phyllosphere warrant further investigation.

These findings suggest that physical properties of mulches or soil may influence the splash kinetics of the droplets generated by rain and the ensuing dispersal of *E. coli* from the fecal source. Plastic mulching is impermeable to water and has a smooth, sleek surface which would promote droplet ricochet, allowing bacteria to travel further distances. Studies have demonstrated this in other pathosystems where plants grown on plastic versus permeable and organic mulches exhibited higher levels of enteric and plant pathogen dispersal and contamination post-irrigation, such as *Salmonella* Typhimurium on tomato (soil, plastic and organic mulch), and *Colletotrichum* (grass and pine mulch), *Phytophthora* (soil, sand and straw mulch) and *Botrytis cinerea* (plastics) on strawberries (Madden and Ellis, 1990; Coelho et al., 2008; Cevallos-Cevallos et al., 2012; Wang et al., 2023). Natural mulches like straw and grass clippings may have the ability to shorten the dispersal of microorganisms by ‘trapping’ them in the mulch matrix, unlike plastic covers which appear to amplify dispersal. Moreover, in previous work, we reported that both synthetic and natural row covers altered soil microclimate in a way that favored the persistence of foodborne pathogens in soil (Micallef et al., 2023). Black plastic is very effective for weed suppression, increasing soil temperature and retaining soil moisture, improving crop yield, although it is economically and environmentally costly and can reduce microbial diversity in the soil compared to organic mulches (Steinmetz et al., 2016). As a result, several biodegradable plastics have become popular. Organic mulches such as straw can also provide the same benefits of plastic while also supporting soil microbial activity (Li et al., 2014). Therefore, while bare ground appears to be the most opportune option for mitigating foodborne pathogen presence in vegetable fields, more research is needed to identify best practices that can balance food safety risk reduction with the benefits provided by the use of row covers.

While we assessed time in relation to feces placement in the field, we also asked whether *E. coli* populations would propagate or remain stable on the plant in the days following the rain event. These data are particularly relevant to determine whether any recommendations to growers on adopting a no-harvest buffer zone are applicable for one or more days after a rain event. *E. coli* levels obtained three days after the rain event exhibited a decline in relation to the first sampling one day after precipitation. This was true for all mulch types, with a decreasing trend in line with observations from one day following precipitation. Indeed, incorporating a two-three day waiting period before harvest following a rain event may be considered a mitigation measure for risk associated with animal intrusion and potential crop contamination. Jeamsripong et al. (2019) tracked *E. coli* populations on Romaine lettuce for 10 days. In this study, bacteria were inoculated directly to lettuce in the form of a fecal slurry and irrigation occurred mid-way through the duration of the experiment. Decline in the *E. coli* population did occur following inoculation and over the entire duration of the study, However, population levels fluctuated, with a particularly noticeable spike following irrigation (Jeamsripong et al., 2019). Bacterial die-off in the phyllosphere of leafy vegetables has been reported to proceed as a function of relative humidity, enteric bacterial species and crop type (Belias et al., 2020). Measures that rely on time intervals from rain events, therefore, should be applied with caution and should not replace the adoption of a no-harvest zone in close proximity to fecal material at the time of harvest.

In our study, the crop stages we evaluated were baby leaf and mature lettuce heads, 10 and 36 days post-field transplantation, respectively. No distinction could be detected in the pattern and degree to which *E. coli* disseminated from a fecal point source to baby leaf and mature lettuce. This suggested that plant age, size of lettuce head and space between lettuce plants (which reduced as plants grew bigger) did not play a major role in modulating dispersal of *E. coli* in the field environment. Limitations of our study, however, need to be taken into consideration when interpreting these findings. Direct comparison of data from the two trials comparing plant age is confounded by the rain event. Rain was simulated in Trial 1A (baby leaf) while a substantial natural rain was evaluated in Trial 1B (mature head). We cannot determine whether simulated rain generated less dispersal, however, we did, in fact, observe augmented levels of *E. coli* transfer in Trial 3, which used baby leaf lettuce and a natural rain event. Trials conducted in Georgia (Trials 2 and 4) also simulated rain and resulted in less dispersal, compared to Maryland. Nevertheless, the data we collected from both natural and simulated rain events are informative and add insight to understanding bacterial transmission in the field in relation to splash.

Another consideration when comparing baby leaf and mature lettuce is the density of the leaf cluster. Cultivar variability in rosette structure, which determines leaf compactness and angle, may be relevant to consider, as more compact or upright lettuce heads may protect the plant meristem and adaxial leaf sides from splash. *E. coli* retrieval on lettuce after contamination from simulated irrigation in the field favored outer leaves over inner leaves (Atwill et al., 2015; Weller et al., 2017). It has been inferred that outer leaves are a preferable site for splash contamination while inner leaves are more suitable for *E. coli* persistence and colonization due to increased nutrients and shelter from harsh environmental conditions (Brandl and Amundson, 2008; Oliveira et al., 2012). The distinction between inner and outer leaves may be less relevant to baby leaf lettuce crops, since the lettuce head is still open at this stage, allowing for splashed droplets to make contact with any part of the plant. Moreover, younger lettuce and kale leaves have been shown to be more supportive to enteric pathogens than older leaves (Brandl and Amundson, 2008; Liu et al., 2022), although this may be dependent on plant cultivar and bacterial serovar (Hunter et al., 2015). Leaf morphological features, which may drive leaf age differences in enteric pathogen association with lettuce (Hunter et al., 2015), were not assessed in this study but are to be expected as plants develop. Nevertheless, we were surprised to see the same degree of dispersal in mature lettuce heads, where the leaf canopy was very dense. One could hypothesize that outer leaves harbored higher *E. coli* levels than inner leaves in mature lettuce, a distinction that was eliminated by our sample processing method, whereby whole lettuce heads were chopped (excluding dead leaves) and sub-samples taken for bacterial quantification. However, whole-head chopping may not vary widely from industry practice. Additional research on plant and leaf morphology paired with bacterial dispersal data could shed light on what cultivar traits and cultivation practices may modulate the degree of bacterial transmission via water splash in the field, to further contribute data that can help inform best harvesting and post-harvest processing practices.

The impetus for this research was to obtain data that would help inform growers in the Atlantic region of the US, with regards to the Food Safety Modernization Act, Produce Safety Rule Subparts I § 112.83 and K § 112.112. These subparts require decision-making at the time of harvest with regards to animal intrusion and the measures that should be taken (including not harvesting) if a crop is identified as potentially contaminated. Studies conducted in other regions of the US suggest that a 1.52 m no-harvest buffer zones is adequate for California (Jeamsripong et al., 2019) and a 0.5 m buffer zone would reduce *E. coli* levels by 98% in New York (Weller et al., 2017). In our study, by the 0.6 m distance, levels of *E. coli* fell below 1 log MPN/sample in all trials except Trial 3. Trial 3 may be considered as the highest risk scenario in our study due to the combination of the highest inoculum and strongest rain event. In this case, data modelling predicted that a 7-log reduction in *E. coli* from levels in feces would be attained by 1.4 m on biodegradable plastic and 0.4 m on straw mulch. Hence the 1.52 m buffer zone that is currently recommended is adequate and may even be excessive, depending on the cropping system. Data on bacterial release under different rain intensities and duration may be needed to further refine this recommendation for the Atlantic region.

In summary, since agricultural conditions, management practices and climate vary greatly by region and have a significant impact on plant growth and microbial dynamics, this study intended to fill a data gap on bacterial dispersal for the Atlantic Region, including the mid- and south Atlantic. Our data analysis and modelling suggested that while risk of contamination of a crop by rain splash-mediated *E. coli* contamination from animal feces did not differ by plant age (baby leaf versus mature lettuce), time of feces placement and distance from the fecal point source were influencing factors. Moreover, absence or type of mulch cover greatly influenced dispersal of *E. coli* mediated by splash. Our data support the recommendation that a no-harvest buffer zone be implemented when feces are detected in fields at the time of harvest, as well as applying an interval between rain and harvest, but the area of the buffer zone should consider recent rainfall, freshness of scat and type of mulch used.

## Data availability statement

The raw data supporting the conclusions of this article will be made available by the authors, without undue reservation.

## Author contributions

AH: Writing – original draft, Methodology, Investigation. CH: Visualization, Methodology, Formal analysis, Data curation, Writing – original draft, Investigation. DK: Writing – review & editing, Investigation. QD: Writing – review & editing, Visualization, Methodology, Investigation, Formal analysis. ZG: Writing – review & editing, Methodology, Investigation. AJ: Writing – review & editing, Methodology, Investigation. AB: Writing – review & editing, Investigation, Data curation. RT: Writing – review & editing, Funding acquisition. TC: Writing – review & editing, Resources, Project administration, Methodology. LD: Writing – review & editing, Supervision, Resources, Project administration, Methodology, Investigation, Funding acquisition, Data curation, Conceptualization. SM: Writing – review & editing, Writing – original draft, Visualization, Validation, Supervision, Resources, Project administration, Methodology, Investigation, Funding acquisition, Formal analysis, Conceptualization.
